# Dispensing apparatus for use in a cued food delivery task

**DOI:** 10.1016/j.mex.2015.11.002

**Published:** 2015-11-10

**Authors:** Menton M. Deweese, Kimberly N. Claiborne, Jennifer Ng, Danika D. Dirba, Hannah L. Stewart, Susan M. Schembre, Francesco Versace

**Affiliations:** The University of Texas MD Anderson Cancer Center, Houston, TX, USA

**Keywords:** Food delivery device, Reward, Cue reactivity, Food availability, EEG-compatible, Obesity

## Abstract

Neurobiological models of obesity postulate that obese individuals have difficulty regulating food intake partly because they attribute excessive salience to stimuli signaling food availability. Typically, human studies that investigate the relationship between brain responses to food-related stimuli and obesity present food cues without subsequent delivery of food. However, in order to identify the brain correlates of cue reactivity, we must record brain responses to food-related cues signaling food availability. Therefore, we have developed a dispensing apparatus for use in a cued-food delivery task in which event-related potentials (ERPs) to food-related images predicting food delivery and images not predicting food delivery can be recorded. Here, we describe a method where:

• The experimental apparatus dispenses an edible item (i.e., a chocolate candy) which may or may not be eaten, or a non-edible control item (e.g., a plastic bead).
• Deposit boxes are available to store uneaten candies and the non-edible control items.
• The dispensing mechanism is capable of recording the exact timestamp when each delivery event occurs (e.g., release from the dispenser, arrival in the receptacle, storage in the deposit box).

## Graphical abstract

Representative diagram of the experimental setup. The laboratory control computer (A) triggers the dispensing device (B) to dispense either a food (candy) or non-food control item in the delivery receptacle (C). The participant may either eat the candy, or deposit the candy or non-food control item in the appropriate deposit box (D). The display of the master laboratory computer (A) is mirrored in the participant laboratory room (E), and displays pictorial stimuli predicting either the food reward or non-food control item. During the experiment, the electroencephalogram (EEG) is being recorded and is displayed on a computer (F) in the laboratory control room. The master computer (A) sends triggers to the EEG computer (F) to allow subsequent time-locking of events. Blue arrows represent information flow from the participant laboratory room to the laboratory control room. Black arrows represent information flow from the laboratory control room to the participant laboratory room.

**Figure:**
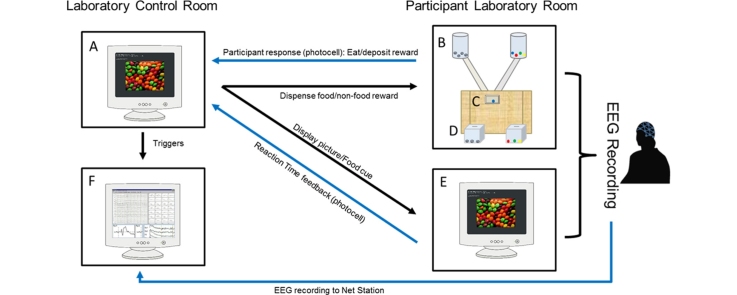

## Method details

In humans, studies investigating the relationship between brain responses to food-related stimuli and obesity typically present visual food cues without subsequent delivery of an edible food reward [1], [2], [3]. Here, we have developed a dispensing apparatus for use in a cued food-delivery task capable of delivering either a food reward (an M&M's^®^ candy) or a non-food control item (e.g., plastic beads), while brain responses to pictorial cues are being recorded via the electroencephalogram (EEG). To build the apparatus, we used items from Med Associates, Inc. (St. Albans, VT, USA) and items available from regular hardware stores. What follows is a brief description of the laboratory setup and dispensing apparatus:

• Two dispensers* hold either food rewards or non-food control items.
• A computer in the laboratory control room (PC1) sends a signal via E-prime^®^ software to display pictorial stimuli on a mirrored computer located in the participant recording room (PC2).
• PC1 simultaneously sends a signal via E-prime^®^ software to one of two dispensers (C1, C2) to dispense either a food or non-food control item.
• A plastic tube directs the food reward/non-food item via a Y-connector from C1/C2 to a metal tray housed in a dispensing receptacle.
• A photo cell is attached to the metal tray to detect when the item is picked up by the participant; the timestamp is recorded by PC1.
• Two deposit boxes are equipped with photo cells to detect when the food reward or non-food item is deposited by the participant; the timestamp is recorded by PC1.
• During the task, the participant's brain responses are being recorded via dense-array EEG, which is monitored on a Macintosh computer in the laboratory control room.

  

* The dispensers used to deliver food rewards and non-food control items are pre-assembled dispensers and require no additional modification or assembly; product information may be found in the “Supply list” section.

## Supply list

### Materials

• 28V* DC to TTL Adapter. Purchased from Med Associates Inc., Model: SG-231. Quantity: 2.
• TTL to 28 V DC Powered Adapter with Coax Connector. Purchased from Med Associates Inc., Model: SG-230RC. Quantity: 3.
• Power Supply 28 V, 1 A DC. Purchased from Med Associates Inc., Model: SG-501. Quantity: 1.
• M&M Dispenser with IR Sentry, Pedestal Mount. Purchased from Med Associates Inc., Model: ENV-702. Quantity: 2.
• Pellet Receptacle, Trough Type, 75–300 mg. Purchased from Med Associates, Inc., Model: ENV-200R7 M. Quantity: 1.
• Dual Feeder “Y” Tube used to connect two feeders to a single receptacle. Purchased from local hardware store.
• Acrylic Cube Ballot Box (6″ × 6″ × 6″). Purchased from Viziflex Seels. Quantity: 2.
• Head Entry Detector. Purchased from Med Associates Inc., Model: ENV-254-CB. Quantity: 3.
• BNC Output cable, 5′ with Fork Lug Termination. Purchased from Med Associates Inc., Model: PHM-155G. Quantity: 5.
• 28 V Daisy Chain Power Cable, 6′ (2 Pin F to 2 Pin F to 2 Pin Male Molex). Purchased from Med Associates Inc., Model: SG-235DC-6. Quantity: 2.
• 25 ft RCA Audio/Video Cable (3 male to 3 male). Purchased from Amazon. Quantity: 2.
• Cuts of plain clear PVC tubing, ¾″ diameter and 3″ length. Purchased from local hardware store. Quantity: 2.
• Cuts of flexible PVC tubing, ¾″ diameter and 13″ length. Purchased from local hardware store. Quantity: 2.
• PVC couplings, 1″ diameter and 1½″ length. Purchased from local hardware store. Quantity: 2.
• PVC 45° elbow, ¾″ opening. Purchased from local hardware store. Quantity: 2.
• Cuts of plain PVC end pipes, ¾″ diameter and 2″ length. Purchased from local hardware store. Quantity: 3.
• PVC female adapter, ¾″ diameter and 1¾″ length. Purchased from local hardware store. Quantity: 4.
• PVC 90° elbow, ¾″ opening. Purchased from local hardware store. Quantity: 1.
• Complimentary pair of magnetic strips, 3½″ × 1″. Purchased from local craft store. Quantity: 1.
• Metal L bracket, 1″ × 1″. Purchased from local hardware store. Quantity: 4.
• Phillips screws with complimentary washers to fit into L bracket. Purchased from local hardware store. Quantity: 4.
• Plywood, ⅕″ thickness, 12″ × 16″. Purchased from local hardware store. Quantity: 3.
• Acrylic-safe glue. Purchased from local craft store.
• Optional: additional Phillips head screws.

*Note*: The supply list includes only non-standard materials. Standard equipment such as a wood rotary saw, PVC cutter, rotary tool, Phillips head screwdriver and measuring tape are assumed to be available.

*28 V is the standard current unit used by Med Associates, Inc. for all power control and interface system equipment.

## Methods

### Assembly of individual apparatus components

#### **Step 1:** delivery receptacle

*Materials*

3 pieces of plywood with ⅕″ thickness, 12″ × 16″ each
6 Phillips head screws, washers
2 1″ L brackets head entry detector
Pellet receptacle

*Procedure*

**Fig. 1:**
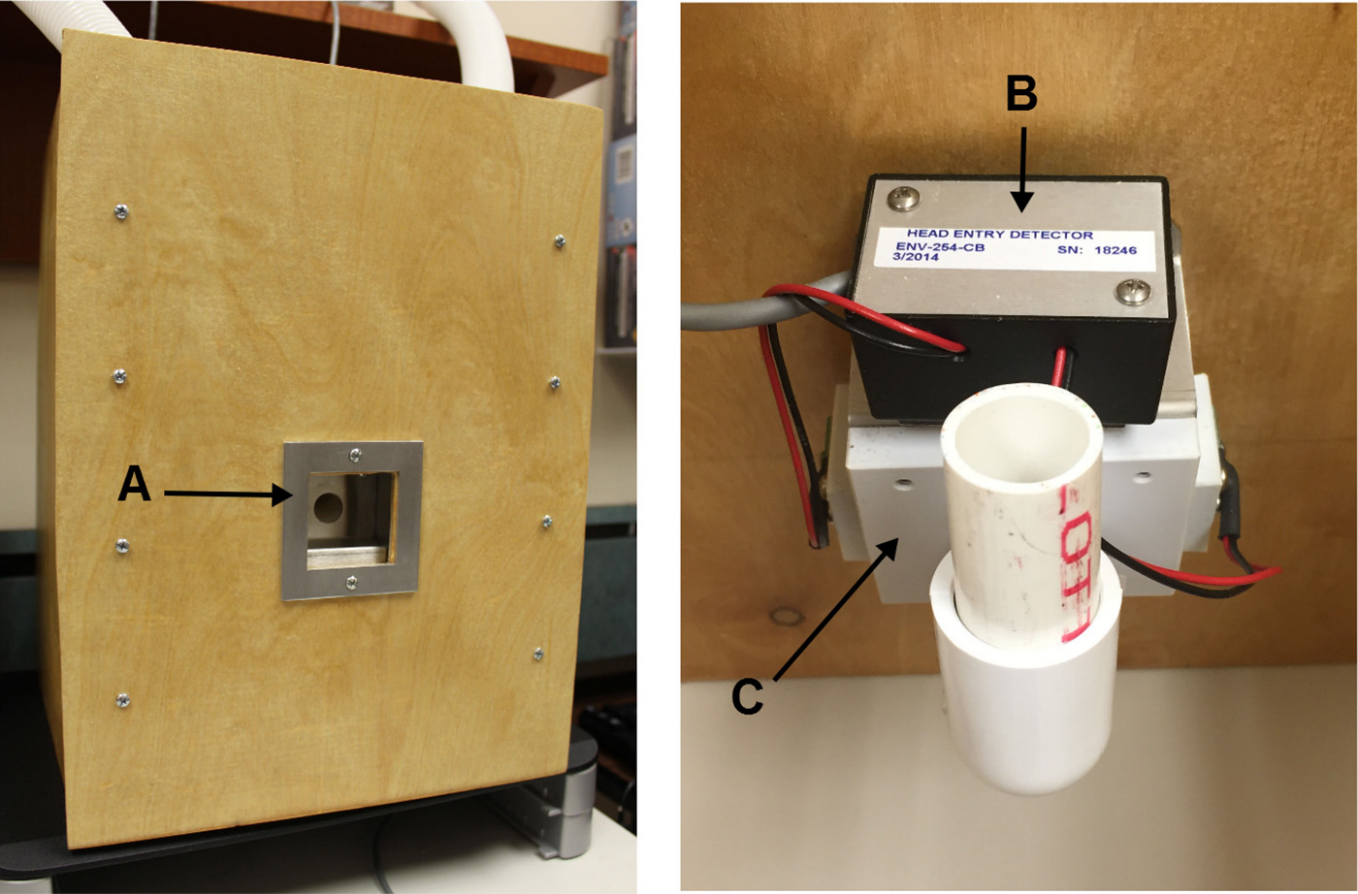
Left: Front view of the assembled wooden delivery receptacle, highlighting the position of the inserted pellet receptacle (A). Right: View from the inside of the delivery receptacle. Head entry detector (B) is mounted directly on top of the secured pellet receptacle (C).

Front panel:

1. Cut a 3″ × 3″ square hole in the center of the plywood to fit the pellet receptacle (Fig. 1A).
2. Insert the pellet receptacle and loosely attach with 2 Phillips head screws.
3. Glue the photocells to the inside of the left/right sides of the pellet receptacle.
4. Tighten the screws of the pellet receptacle, to secure it in place.
5. Inside the receptacle, glue the bottom of the head entry detector (Fig. 1B) to the top of the pellet receptacle (Fig. 1C).

Side panels:

1. Align one of the side panels to the front panel, lengthwise. Secure in place using Phillips head screws, washers, and two 1″ L brackets approximately 3¼″ from the top and another 3¼″ from the bottom.
2. Repeat step 2 with the other side.

#### **Step 2**: Y-connector

*Materials*

1 Dual feed Y connector
2 cuts of plain clear PVC tubing, ¾″ diameter and 3″ length
2 cuts of flexible PVC tubing, ¾″ diameter and 13″ length
2 PVC couplings, 1″ diameter and 1 ½″ length
2 PVC 45° elbow, ¾″ opening
3 cuts of plain PVC end pipes, ¾″ diameter and 2″ length
4 PVC female adapter, ¾″ diameter and 1¾″ length
1 PVC 90° elbow, ¾″ opening

*Procedure*

**Fig. 2:**
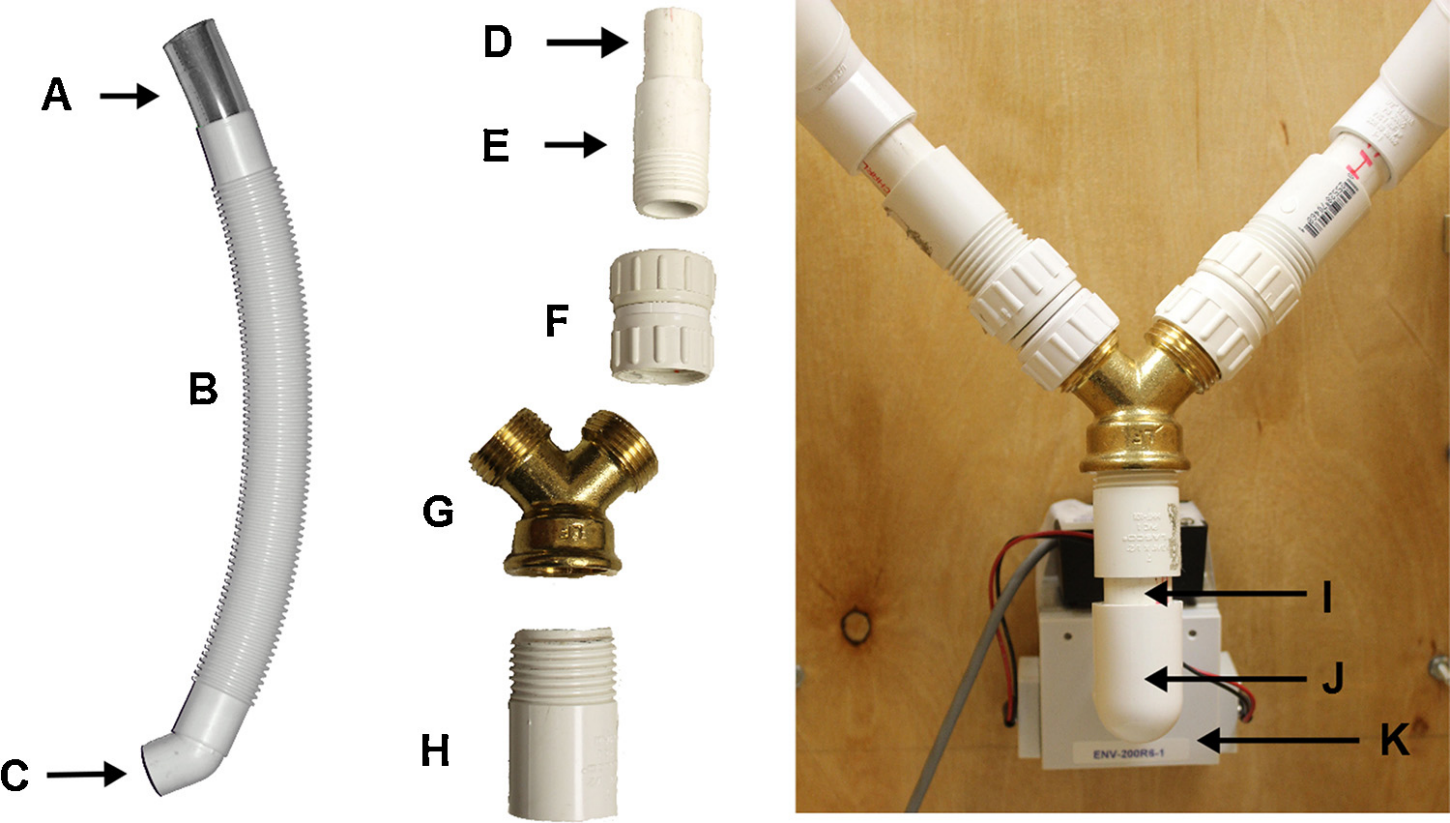
Left: A step-by-step layout of the pieces needed to assembly the two branches of the Y-connector. Note that duplicate components A–F are necessary to construct the additional branch of the Y-connector, and are not pictured here. Right: Components A–H are joined together via components I–J, connecting to the head entry detector on the inside of the delivery receptacle.

1. Connect the fittings as follows to create Tube 1:
  a. Clear PVC tube (Fig. 2A) to flexible PVC tube (Fig. 2B).
  b. Flexible PVC tube to PVC 45° elbow (Fig. 2C).
  c. PVC 45° elbow to the plain PVC end pipe (Fig. 2D).
  d. Plain clear PVC end pipe to the PVC female adapter (Fig. 2E).
  e. PVC female adapter to the PVC coupling (Fig. 2F).
  f. PVC coupling to the Dual feed Y connector (Fig. 2G).
2. Repeat Step 1, a–f for the remaining components to create Tube 2.
3. Connect dual feed Y connector (Fig. 2G) to the PVC female adapter (Fig. 2H).
4. Connect the PVC female adapter to the plain PVC end pipe (Fig. 2I).
5. Connect plain PVC end pipe to PVC 90° elbow ¾″ opening (Fig. 2J).
6. Connect PVC 90° elbow ¾″ opening to pellet receptacle (Fig. 2K).

#### **Step 3:** deposit boxes

*Materials*

2 acrylic ballot boxes 2 head entry detectors.
4 cuts of complimentary magnetic strips, 1¾″ × 1″.
Optional: additional Phillips screws.

*Procedure*

**Fig. 3:**
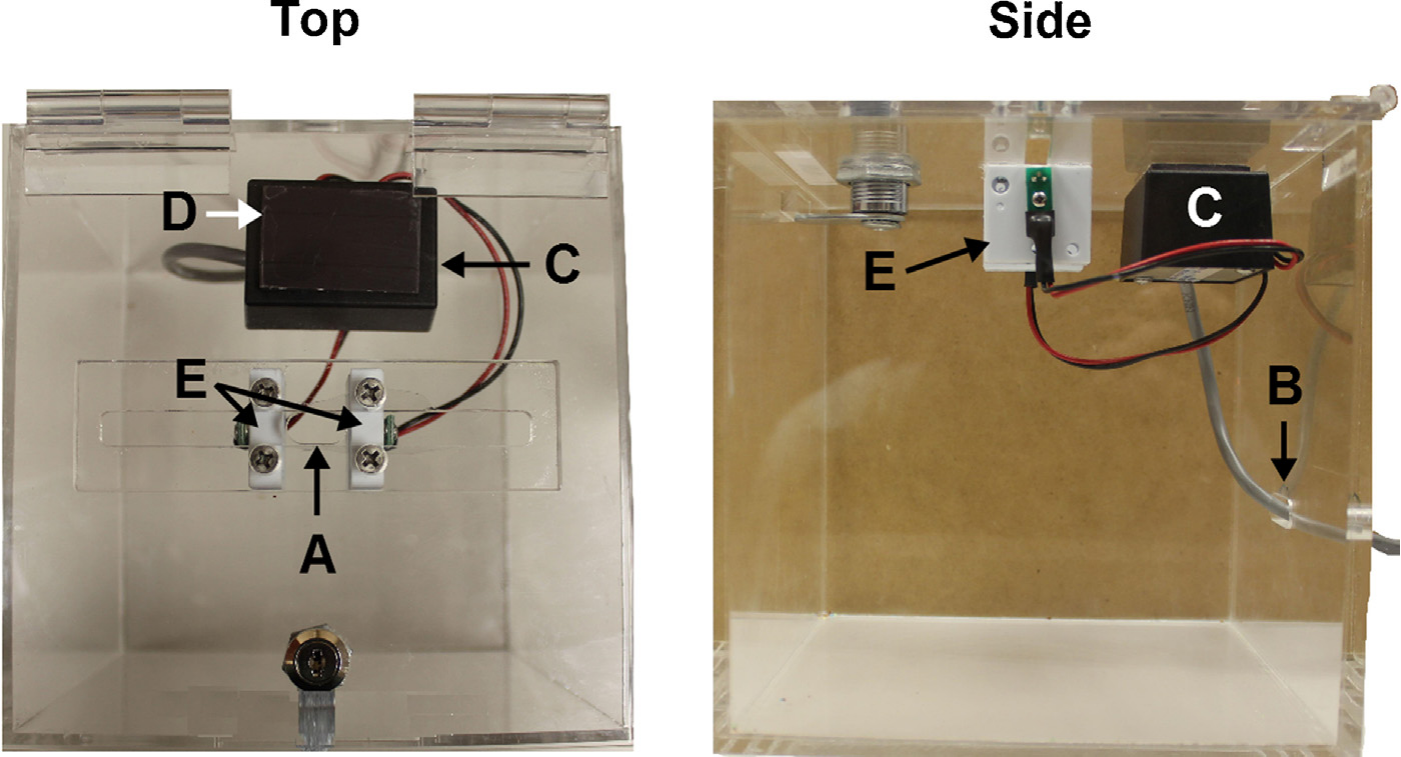
Top (left) and side (right) view of the deposit box for the food reward/non-food item.

Deposit box 1:

1. Using a rotary tool, grind the center of the ballot box slot to form a ¾″ × ⅕″ hole, large enough for food reward (Fig. 3A).
2. Using a rotary tool, drill a hole in the back of the ballot box to feed the wires from the head entry detector, approximately 1″ × ⅕″ (Fig. 3B).
3. Place head entry detector (Fig. 3C) inside of the ballot box and feed the wire through the hole (Fig. 3B).
4. Glue a 1 ¾″ × 1″ magnetic strip to the inside lid of the box approximately 1″ away from the slot in the top of the ballot box (Fig. 3D).
5. Glue the complimentary 1¾″ × 1″ magnetic strip to the bottom of the head entry detector (Fig. 3C).
6. Using acrylic-compatible glue, glue the white photo cells (Fig. 3E) from the head entry detector (Fig. 3C) on either side of the slot hole (Fig. 3A) on the underside of the top of the ballot box. Optional: secure with Phillips head screws.

Deposit box 2:

1. Repeat steps 1–6 from Deposit box 1.

### Apparatus assembly

#### **Step 4:** connect Deposit box 1 and Deposit box 2 to power source and PC1

*Materials*

Deposit box 1
Deposit box 2
Box 1 (B1)
Box 2 (B2)
2 RCA Audio/Video cable (R1, W1, R2, W2)
PC1
PC2
DIS1
DIS2
Black Receptacle Box (RB)
Delivery Receptacle
28 V power source box, wall plug

*Procedure*:

Refer to Fig. 6 for a full connectivity diagram.

#### **Step 4a:** Deposit box 1

1. The output cable extending from the Head Entry Detector (see Fig. 3B) in Deposit box 1 is connected to the input port of Box 1.
2. Box 1 TTL ground/output is connected to the red (R1) RCA Audio/Video cable, which is then connected to PC1 in laboratory monitoring.
3. The 28 V port on Box 1 is connected via the split cable from Box 2 (see below).

#### **Step 4b:** Deposit box 2

1. The output cable extending from the Head Entry Detector in Deposit box 2 is connected to the input port of Box 2.
2. Box 2 TTL ground/output is connected to the red (R2) RCA Audio/Video cable, which is then connected to PC1 in laboratory monitoring.
3. Connect to the power source:
  a. 28 V wire on Box 2 is split:
    i. One branch is connected to the 28 V port on Box 1
    ii. One branch is connected to the 28 V port on DIS2
      1. The 28 V branch connecting to DIS2 is split further and connects:
        a. to the 28 V input of DIS1.
        b. to the 28 V input on the black receptacle box (see Step 4c, 1).

#### **Step 4c:** receptacle box, power source

**Fig. 4:**
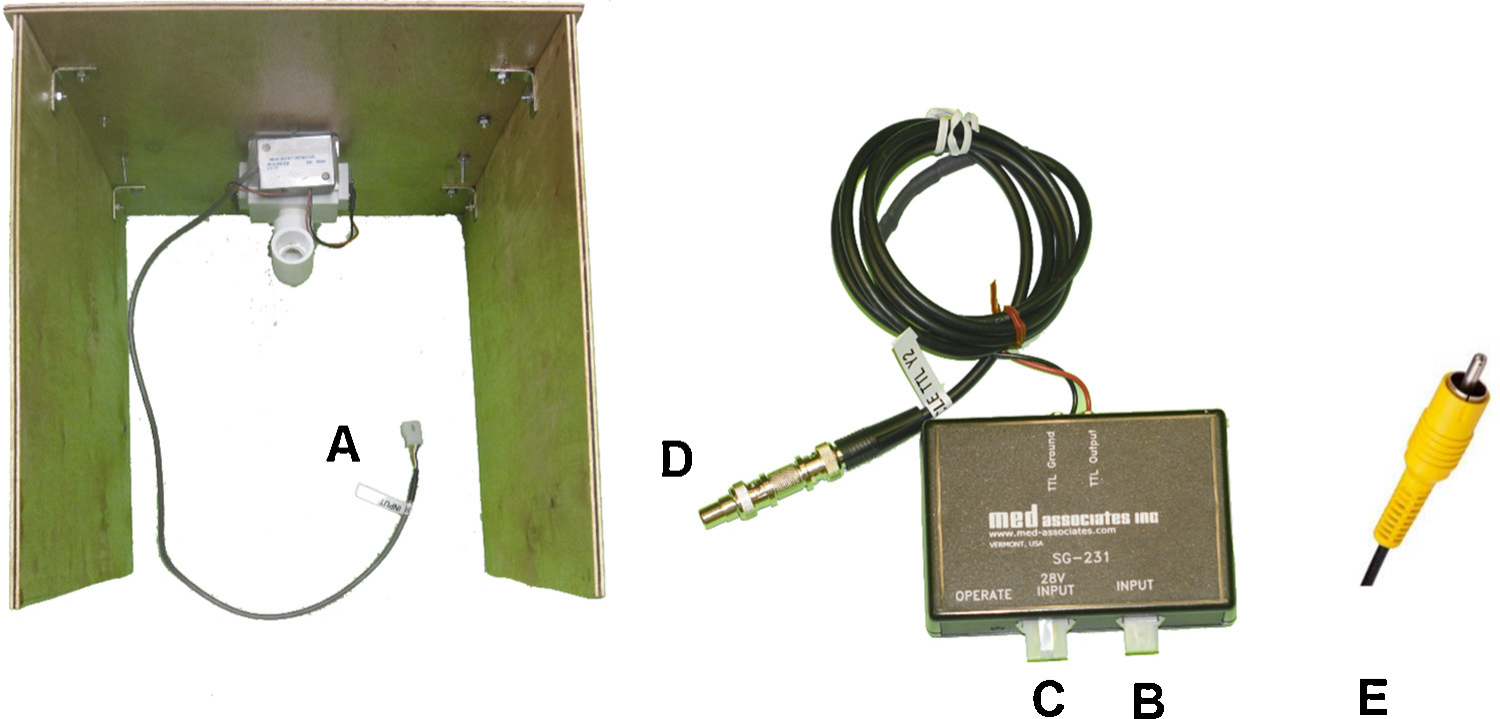
Layout of the components necessary to connect the head entry detector port (A) to both the power source (B and C) and master computer in laboratory monitoring (D and E).

1. The output cable extending from the Head Entry Detector port (Fig. 4A) mounted on the wooden receptacle is connected to the input port of black receptacle box RB (Fig. 4B).
2. The 28 V input port of the RB (Fig. 4C) is connected directly to the power supply box and to DIS2, completing the voltage circuit.
3. The TTL ground/output wires are connected to Y2 (Fig. 4E), which is connected to PC1 in laboratory monitoring.

#### **Step 5:** connect dispensers (Cylinder 1, Cylinder 2) to DIS1 and DIS2, then to PC1 in laboratory monitoring

*Materials*

Cylinder 1
Cylinder 2
DIS1
DIS2
2 RCA connector cables
PC1

*Procedure*

Cylinder 1 (C1):

1. The C1 cable is connected to the output port of DIS1.
2. The 28 V input port is connected to the 28 V port on DIS 2.
3. The TTL ground/input wire on DIS1 is connected to the white (W1) RCA Audio/Video cable, which is connected to PC1 in laboratory monitoring.

Cylinder 2 (C2):

1. The C2 cable is connected to the output port of DIS2.
2. The 28 V power supply is split (see Step 4b, 3).
3. The TTL A/V input on DIS2 is connected to the white (W2) RCA connector, which is connected to PC1 in laboratory monitoring.

#### **Step 6:** connect dispensers to delivery receptacle via Y-connector

*Materials*

C1
C2
Tube 1
Tube 2
Y-connector
Delivery receptacle

*Procedure*

**Fig. 5:**
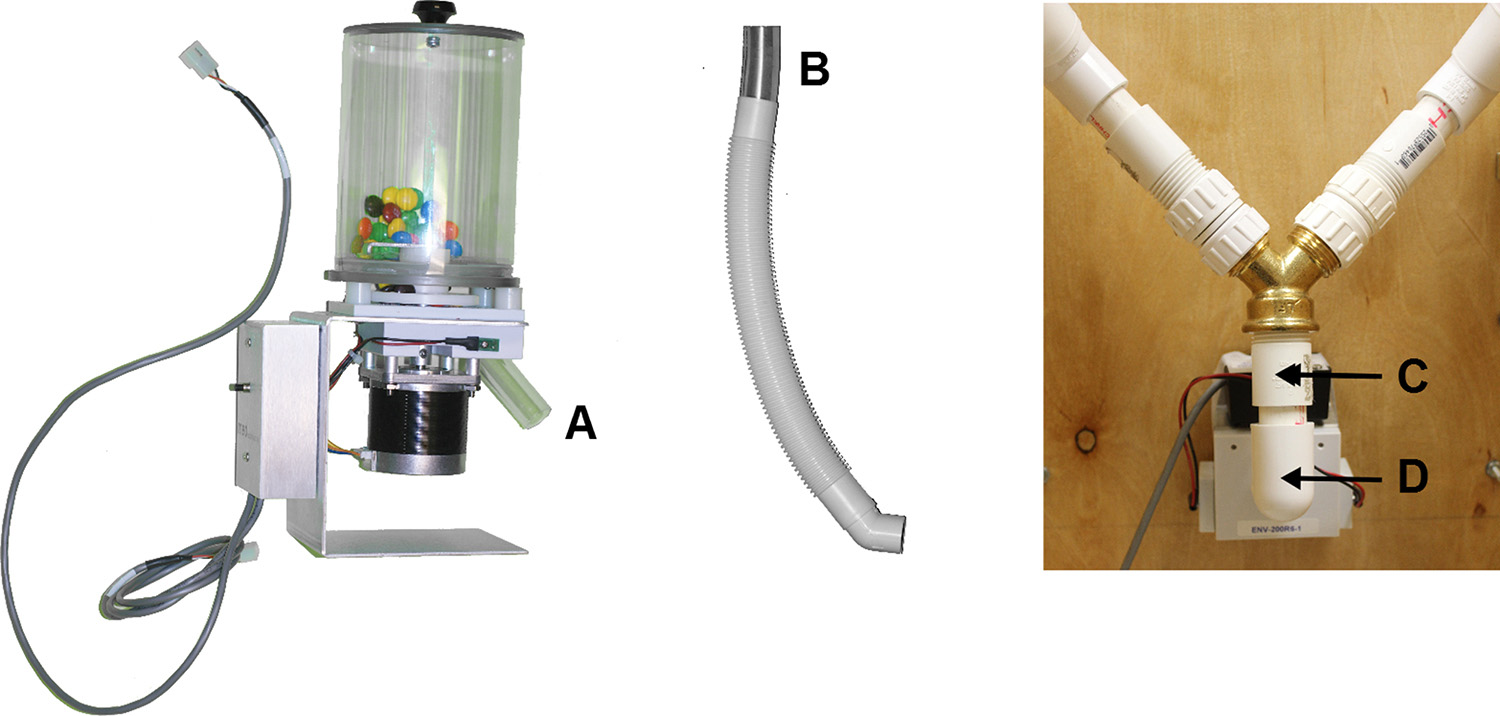
Components necessary to connect the food reward/non-food item dispenser to the delivery receptacle.

1. Using the Y-connector assembled in Step 2, connect the dispenser output of C1 (Fig. 5A) to the clear PVC tube (Fig. 5B). Similarly, connect the end of the clear PVC tube of the remaining tube to the dispenser output of C2.
2. Connect the base of the fully assembled Y-connector (Fig. 5C) to the L-shaped PVC connector on the inside of the delivery receptacle (Fig. 5D).

## Dispenser activation via E-prime^®^

Communication with the parallel port was controlled by E-prime^®^ software. E-prime^®^ “Inline” scripts were used to send event triggers to the dispensing devices. The parallel port address was expressed in hexadecimal notation, and all pins of the parallel port were set to zero at the start of the program using an Inline script (WritePort &H378, 0). In the trials in which an item was delivered, an Inline script activated one parallel port pin for 50 ms:

WritePort &H378, 2
sleep 50
WritePort &H378, 0

Different output pins were used to activate each dispenser (i.e., pin 1 or pin 2). To receive inputs from the devices via the parallel port, the parallel port was added as an “input device” to the E-prime^®^ “slide” object presented when inputs from the participant were expected. “If…then” loops within Inline scripts placed after each slide object controlled the behavior of the E-prime^®^ program. For example, following item delivery, a transparent slide object was “superimposed” onto the image on the screen, declaring the parallel port as an input device. The duration of the slide object was set to “infinite” to allow for variable reaction times to retrieve the item from the receptacle. An “If…then” loop in an Inline script monitored the parallel port pin connected to the photocell in the receptacle. Once the signal on that pin was received (e.g., an M&M^®^ candy was delivered to the receptacle), the “If…then” loop triggered the next appropriate slide (e.g., a reminder to take the appropriate action: eat or deposit the candy), and the relevant information (e.g., response time) was logged in the E-prime^®^ data file. To avoid potential infinite loops and allow for flexible behavior in case of unexpected events, the Inline script also monitored and managed inputs from the keyboard in the control room (e.g., to allow the researcher to force the start of the next trial if the participant dropped the candy) and the pins of the parallel port associated to the deposit boxes.

## Response recording and timing accuracy

Each dispensing device (see Fig. 5A) was pre-fit with a sensitive infrared photocell located within the delivery tube of the dispensing device. By recording the output of the photocell receptor (via 28 V DC input card), it is possible to estimate the time it takes for an item to be released from the dispenser following an E-prime^®^ trigger event. The temporal precision of item delivery from the dispensing device to the pellet receptacle may also be estimated by recording the response time of photocell activation within the delivery tube and subsequent activation of the photocell within the pellet receptacle. Upon delivery of a food reward, participants may either eat the candy or place it in the deposit box; non-food control items are deposited in the designated deposit box. Deposit boxes for both food rewards and non-food control items are outfitted with photocell receptors (see Step 3) that record the event timestamp of deposited items (via PC1). Based on the needs on an individual experiment, it is possible to record the temporal duration of individual events (i.e., trigger initiation in E-prime^®^ to dispenser, dispenser to receptacle, receptacle to deposit box) or the summation of all events (i.e., trigger initiation to deposit).

## Patch panel wiring circuit

**Fig. 6:**
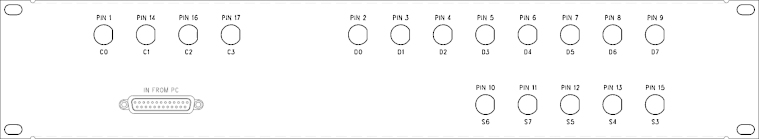
Schematic example of “patch panel” input/output pin layout suitable for the computer-parallel port interface.

To allow for an interface between the computer parallel port and the various devices in the participant laboratory recording room, a “patch panel” was built. Each input and output pin of the parallel port was mapped on the patch panel (see Fig. 6) and BNC connectors and adapters were used in combination with RCA cables.

## Additional information

**Fig. 7:**
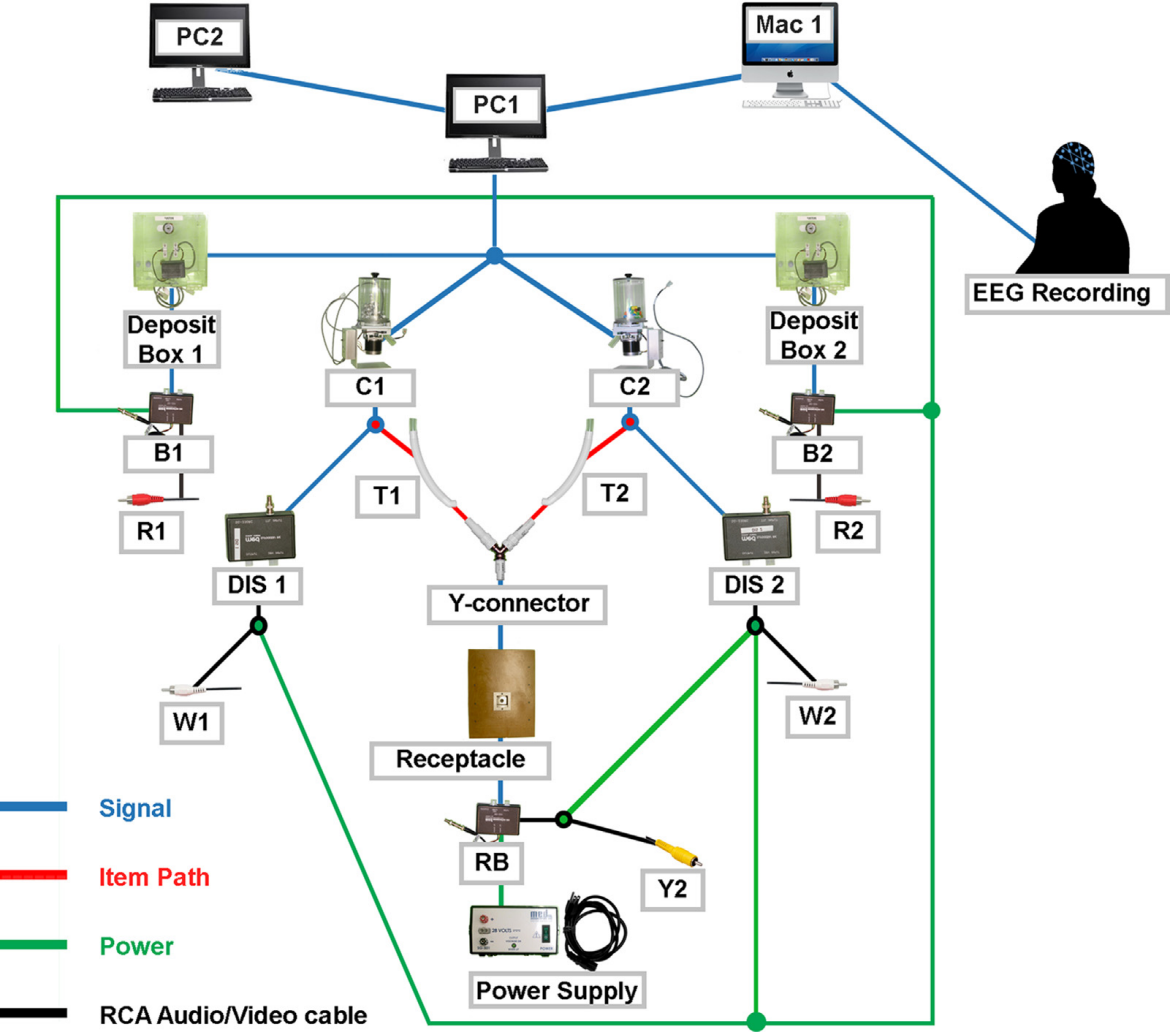
Connectivity diagram of power, signal, item, and audio/visual (A/V) flow through individual components of the food reward/non-food item delivery apparatus. Blue arrows represent signal flow from the master computer (PC1), green arrows represent electrical power flow, red arrows represent the path of the food reward/non-food item through the dispensing/delivery apparatus, and black arrows represent A/V connections to PC1. *Note*: Power/signal flow details of the internal components of the delivery receptacle are further elaborated in Fig. 4 (*).

**Fig. 8:**
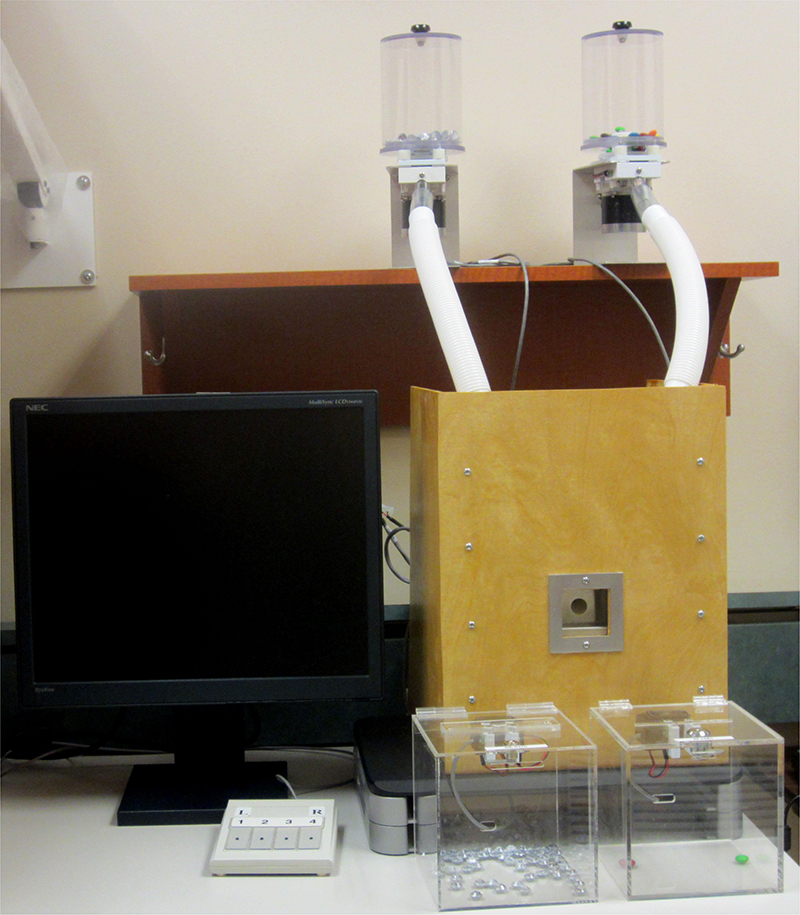
Fully assembled food/non-food delivery apparatus.

A full diagram of signal flow, power connection, delivery item path, and RCA audio/video connection to the master computer (PC1) is illustrated in Fig. 7. The full experimental set-up is depicted in Fig. 8.

The food reward/non-food item delivery device has been implemented in our laboratory using E-prime software (version 2.0.8.74; PST Inc., Pittsburgh, PA). E-prime has been configured to deliver pictorial cues predicting the food reward or non-food control item, the display of which is mirrored onto PC2 in the participant laboratory room (see Fig. 8). Communication and subsequent delivery of the food reward/non-food control items occurs between PC1 and C1/C2, via the parallel port. Reaction times and behavioral responses associated with eating/depositing the food reward/non-food control items are also recorded using E-prime software.

## Funding

This work was supported by start-up funds provided to Francesco Versace by The University of Texas MD Anderson Cancer Center. Menton M. Deweese was supported by a cancer prevention fellowship through The University of Texas MD Anderson Cancer Center, Cancer Prevention Research Training Program, funded by the National Cancer Institute grant (R25T CA057730), Shine Chang, Ph.D., Principal Investigator.

## Conflict of interest

All the authors report no biomedical financial interests or potential conflicts of interest.
